# A late Holocene pollen record from proglacial Oblong Tarn, Mount Kenya

**DOI:** 10.1371/journal.pone.0184925

**Published:** 2017-09-19

**Authors:** Colin J. Courtney Mustaphi, Konrad Gajewski, Rob Marchant, Gunhild Rosqvist

**Affiliations:** 1 York Institute for Tropical Ecosystems, Environment Department, Wentworth Way, University of York, York, United Kingdom; 2 Department of Archaeology and Ancient History, Uppsala Universitet, Uppsala, Sweden; 3 Laboratory for Paleoclimatology and Climatology, Department of Geography, Environment and Geomatics, University of Ottawa, Ottawa, Canada; 4 Institutionen för naturgeografi, Stockholms Universitet, Stockholm, Sweden; Centre National de la Recherche Scientifique, FRANCE

## Abstract

High-elevation ecosystems, such as those on Mount Kenya are undergoing significant changes, with accelerated glacial ice losses over the twentieth century creating new space for alpine plants to establish. These ecosystems respond rapidly to climatic variability and within decades of glacial retreat, Afroalpine pioneering taxa stabilize barren land and facilitate soil development, promoting complex patches of alpine vegetation. Periglacial lake sediment records can be used to examine centennial and millennial scale variations in alpine and montane vegetation compositions. Here we present a 5300-year composite pollen record from an alpine tarn (4370 m asl) in the Hausberg Valley of Mount Kenya. Overall, the record shows little apparent variation in the pollen assemblage through time with abundant montane forest taxa derived and transported from mid elevations, notably high abundances of aerophilous *Podocarpus* pollen. Afroalpine taxa included *Alchemilla*, *Helichrysum* and *Dendrosenecio*-type, reflecting local vegetation cover. Pollen from the ericaceous zone was present throughout the record and Poaceae percentages were high, similar to other high elevation pollen records from eastern Africa. The Oblong Tarn record pollen assemblage composition and abundances of *Podocarpus* and Poaceae since the late Holocene (~4000 cal yr BP-present) are similar to pollen records from mid-to-high elevation sites of nearby high mountains such as Mount Elgon and Kilimanjaro. These results suggest a significant amount of uphill pollen transport with only minor apparent variation in local taxa. Slight decreasing trends in alpine and ericaceous taxonomic groups show a long-term response to global late Holocene cooling and a step decrease in rate of change estimated from the pollen assemblages at 3100 cal yr BP in response to regional hydroclimatic variability. Changes in the principal component axis scores of the pollen assemblage were coherent with an independent mid-elevation temperature reconstruction, which supported the strong influence of uphill pollen transport from montane forest vegetation and association between temperatures and montane vegetation dynamics. Pollen accumulation rates showed some variability related to minerogenic sediment input to the lake. The Oblong Tarn pollen record provides an indication of long term vegetation change atop Mount Kenya showing some decreases in local alpine and ericaceous taxa from 5300–3100 cal yr BP and minor centennial-scale variability of montane taxa from mid elevation forests. The record highlights potentials, challenges and opportunities for the use of proglacial lacustrine sediment to examine vegetation change on prominent mountain massifs.

## Introduction

Evidence of glaciation on Mount Kenya (5199 m asl) suggests ice has likely persisted >100,000 years [1–3] and during the Last Glacial Maximum of the Late Pleistocene (~21000 yr BP), covered up to 200 km^2^, reaching as low as 3200–3300 m asl down the mountain [4–6]. Glacial extents varied through the post-glacial period, and glaciers significantly retreated during the twentieth century [6–8] with accelerated loss in recent decades [9–11]. Easterly regional atmospheric circulation transports moisture from the Indian Ocean to the interior of Kenya and supplies Mount Kenya, supporting wet montane forests and glacial ice at peak elevations [12–13]. Precipitation is highest on the southeastern slopes on the windward side with a slight rain shadow effect on the northwest slopes. The glaciers northwest of the mountain peak are the least extensive [6, 14–15] due to the orographic rain shadow caused by the predominant southeasterly winds over the mountain and equilibrium line altitude varies with aspect [16].

Glacier retreat in the current century could potentially induce novel hydroseral transitions in the numerous proglacial tarn lakes [17] and change the alpine vegetation. These are the headwaters for many montane streams flowing into lowland springs and river systems crucial to human populations, wildlife and socio-ecological resilience [18–20] although the glaciers themselves are not significant regional water reservoirs [21]. Mount Kenya glaciers are controlled by a complex interaction between temperature, precipitation, and radiation, with cloud cover being an important influence [22]. The impact of these environmental changes on to the unique vegetation assemblages of the alpine and ericaceous zone is little understood, and there is little data about the long-term response of these systems to Holocene climate variability. Given the strong spatial constraints on the vegetation zones around the mountain, understanding the long-term response of these plant assemblages, which contain many endemic taxa, to climate changes is a crucial question.

In recent decades, the changing thermal regime of the high elevation region on the mountain has led to shifts in alpine vegetation distributions [23–25]. Stabilization of soils through reduced frost action and establishment of finer grained organic soils by microbial communities and pioneer species leads to persistent plant communities >50 years after ice retreat [24–28]. Soil formation is slow and phosphorus and potentially pH limitations maintain low soil microflora in the high alpine valleys [27, 29]. The continued ablation of alpine ice caused by thermal and hydroclimatic changes associated with global climate change will lead to continued changes in vegetation distributions and increase ecological sensitivity to accidental introductions as seen on other isolated mountains in eastern Africa [30].

Vegetation records from cold regions are often sensitive to climatic change, and a number of studies have examined the vegetation change and vegetation histories on Mount Kenya (Table 1). Since the early botanical surveys of Mount Kenya, the alpine zone has been recognized as a center of high species endemism [31–33]. Studies range from repeated surveys of air photographs [34] or permanent vegetation plots [24–26, 28] to sediment-based studies that examine change since the Pleistocene [35–41]. To date, there is one published pollen record (Hobley Valley mire [42]) from the alpine zone (~4000–5000 m asl [43]) located in an unglaciated valley on the windward mountainside; however this site has few geochronological age determinations. High-elevation lake sediment studies of Mount Kenya have focused on glacier histories [8, 44–45], sediment isotopes [46–48] and carbon cycling [40]. Here we present new results from a pollen analysis of radiocarbon-dated sediment cores collected from Oblong Tarn, a proglacial lake on Mount Kenya. These data provide information on vegetation changes in the alpine and ericaceous zones as well as documenting the uphill transport of pollen from lower Afromontane elevations. This new high-elevation pollen record is compared to reconstructed temperature variability on Mount Kenya and other paleoenvironmental data from neighbouring highlands in eastern Africa to examine montane vegetation changes since the termination of the African Humid Period to present.

**Table 1:** Published studies of vegetation change on Mount Kenya.

Location	Distance from peak (km)	Elevation (m asl)	Study	Time interval (cal yr BP)	Focus	References
Tyndall and Lewis Valleys	0.5	4500	botanical surveys	CE 1958–1984	vegetation cover	Spence 1989 [34]
Tyndall Valley	0.5	4500	repeat botanical plots	CE 1992–2002	vegetation cover	Mizuno 2005a, 2005b [25–26]
	0.5	4500	repeat botanical plots	CE 1996, 2011	vegetation cover	Mizuno & Fujita 2014 [28]
Simba Tarn	1.5	4595	lake sediments	8500-present	diatoms, O isotopes	Barker et al. 2001 [47]
Oblong Tarn	1.2	4370	lake sediments	5300-present	pollen, LOI	This study
Hausberg Tarn	1.3	4360	lake sediments	4000–1000	O isotopes	Barker et al. 2001 [47]
Teleki Valley	2	4300	pedology	1980s CE	soil microflora	Mahaney & Boyer 1987 [99]
Hobley Valley mire	2.5	4265	mire peat sediments	5500-present	pollen	Perrott 1982 [63]
Small Hall Tarn	4.5	4070	lake sediments	14000-present	O isotopes, diatoms, pollen	Barker et al. 2001 [47]
			lake sediments	14000-present	C isotopes, biomarkers	Street-Perrott et al. 2004, 2007 [40, 48]
Rutundu Lake	22	3081	lake sediments		pollen	Coetzee 1967 [61]
			lake sediments		diatoms	Barker et al. 2000 [98]
			lake sediments	>35000-present	pollen, biomarkers, grass cuticles	Ficken et al. 2002 [100]
			lake sediments	38300-present	pollen, grass cuticles, C isotopes	Wooller et al. 2003 [39]
			lake sediments	>35000-present	C isotopes, biomarkers	Street-Perrott et al. 2004, 2007 [40, 48]
			lake sediments	18500-present	biogenic Si	Barão et al. 2015 [101]
Rumuiku swamp	28	2160	lake sediments	27000-present	pollen, microscopic charcoal	Rucina et al. 2009 [41, 67]
Sacred Lake	33	2350	lake sediments	>40000-present	pollen	Coetzee 1964, 1967 [35, 61]
			lake sediments	40000-present	C isotopes, biomarkers	Huang et al. 1999 [37]
			lake sediments	2000-present	leaf waxes	Konecky et al. 2014 [102]
Quarry near Chogoria	35	~1700	geobotany	Not known	charred wood encased in lightly welded felsic tuff	Collection in Geology, NMK
Lake Nkunga	43	1830	lake sediments	42000-present	C isotopes, biomarkers	Ficken et al. 1998 [36]
			lake sediments	42000-present	pollen	Olago et al. 1999 [90]
Kiluli swamp	58	1390	lake sediments	4000-present	plant macrofossils	Olago et al. 2003 [38]

Studies of Late Pleistocene and Holocene vegetation changes on Mount Kenya, ordered by distance from Batian peak (5199 m asl). Acronyms: CE, Common Era; NMK, National Museums of Kenya, Nairobi; yr BP, calibrated radiocarbon years before present (CE 1950).

### Study site

**Fig 1:**
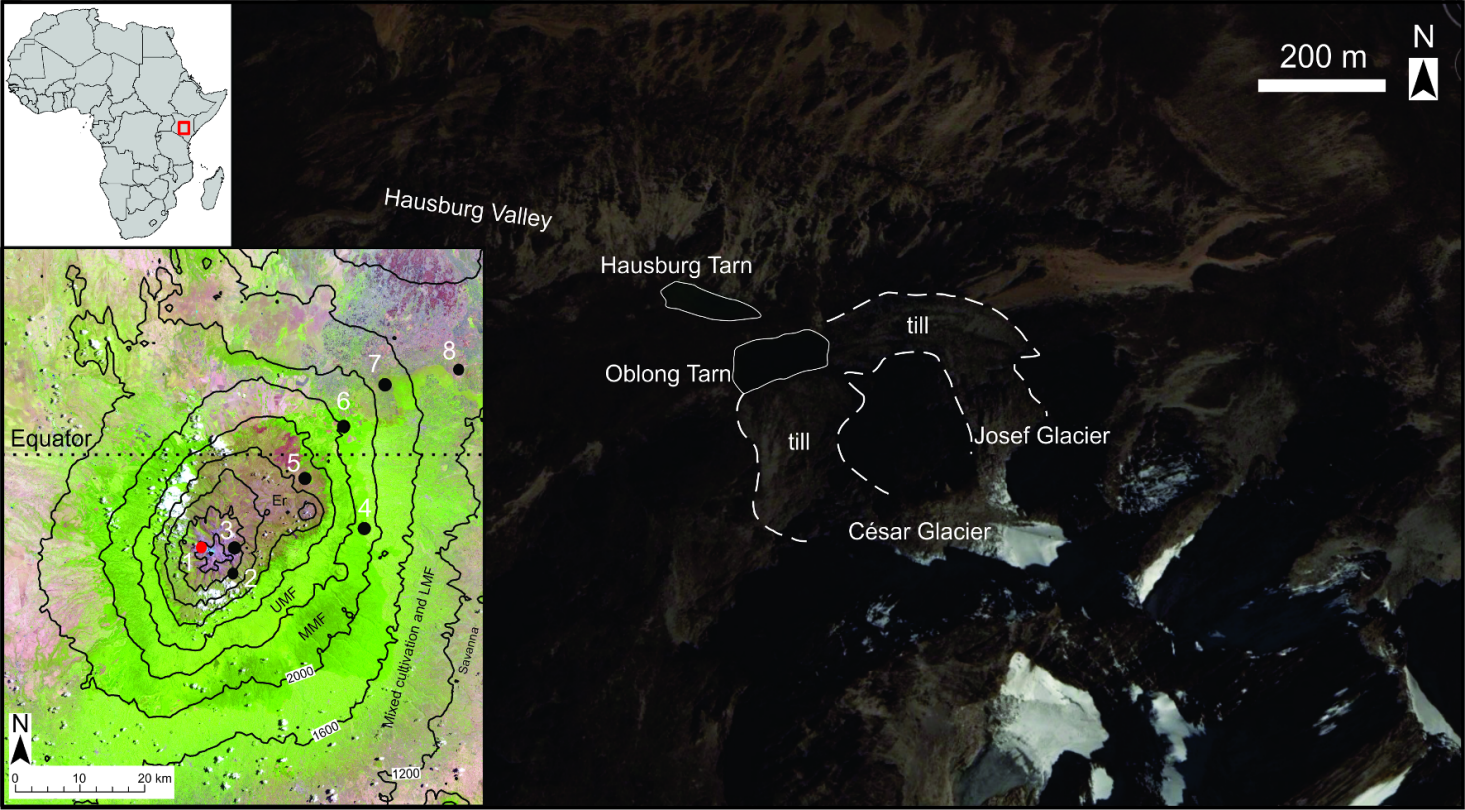
Study location and other published studies mentioned in text. Location map in eastern Africa (upper left inset) and Mount Kenya (lower left inset) showing: 1) Oblong Tarn (this study, red circle), 2) Hobley Valley mire [63], 3) Small Hall Tarn [47], 4) Rumuiku swamp [41], 5) Lake Rutundu [61], 6) Sacred Lake [35, 61], 7) Lake Nkunga [36], 8) Kiluli Swamp [38]. Lower left inset: Landsat true colour image showing Mount Kenya (scene LC81680602014258LGN00; 15 September, 2014; Data available from the U.S. Geological Survey) with 400 m elevation contours (black lines) and summarised vegetation zones: mixed savanna, mixed cultivations and lower montane forest (LMF), moist montane forest (MMF), upper montane forest (UMF), ericaceous zone (Er) and alpine (vegetation zone boundaries are diffuse and not delimited here). Main figure showing headwater lakes (white outlines) of the Hausburg Valley with Oblong Tarn study site near the recessional tills (dashed outlines) of Josef and César Glaciers. (Image date 14 January 2015; Google Earth/DigitalGlobe, 2015).

Mount Kenya (5199 m asl, Fig 1 inset) is an isolated massif of an extinct volcano of Tertiary age that has been largely inactive during the Quaternary [49–50] and has maintained an Afroalpine floristic zone since the Late Pliocene [3, 51]. Bedrock consists of basalts, phonolite, kenyte, agglomerates, trachyte and syenite [49–51].

**Fig 2:**
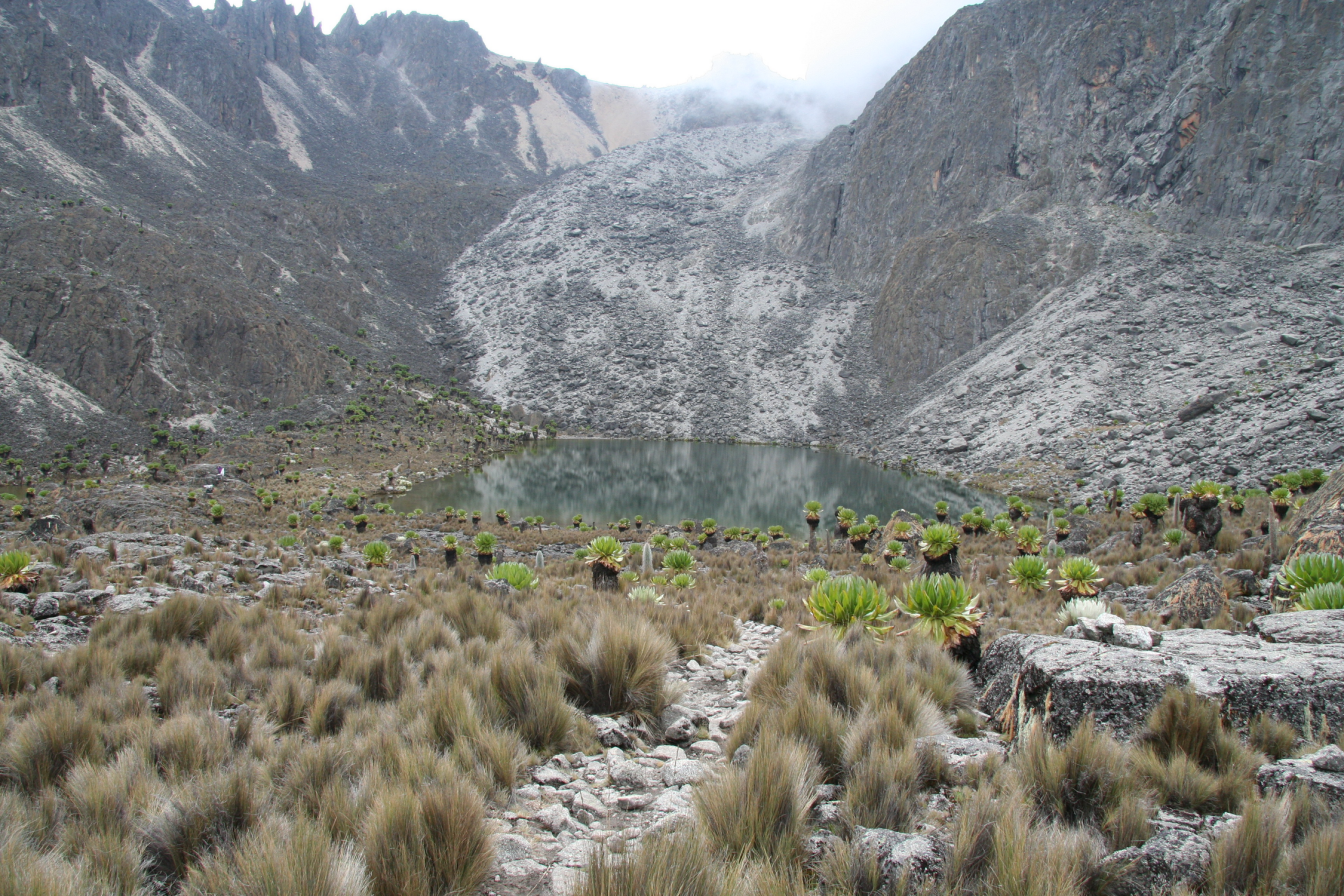
Study site. Photograph of Oblong Tarn from the west shore facing east to till deposits from the retreat of Josef Glacier and showing the local patchy alpine vegetation. Photograph credit: Hilde Eggermont (Ghent University) taken 25 August 2010.

Oblong Tarn (0.145306°S, 37.301707°E; 4370 m asl) is a high elevation proglacial lake on the northwestern (lee) side of Mount Kenya located near recessional till deposits from César and Josef Glaciers at the upper reach of the Hausburg Valley (Figs 1 and 2). The head of the valley has steep slopes within the deglaciated valley and is characterized by bunchgrasses and barren patches of gravel, cobbles and boulders and some mosses (Fig 2). Poaceae, Asteraceae (including large *Dendrosenecio*), Cyperaceae, *Lobelia*, *Alchemilla*, and *Helichrysum* commonly occur in patches where shallow soils have developed.

The cold climate has a strong diurnal thermal, radiation, wind and moisture regime. At night, surface winds flow downhill frequently clearing the night skies and causing cloudiness in confluence zones down the mountain. Early daytime circulation is uphill and carries moisture upward causing clouds at high elevations [52]. Weather conditions, estimated using a lapse rate of 0.6°C per 100 m elevation relative to the Nanyuki meteorological station (0.03° N, 37.02° E) [28] suggest a minimum annual temperature of -4°C. Meteorological measurements at Lewis Glacier from 2009–2012 show a mean temperature of -1°C (1σ = 1.4°C) and an average daily temperature range of approximately -7 to 7°C, low atmospheric pressure (mean = 570 hPa) [22]. Warmest monthly temperatures occur in March-June and coldest from December-February. Precipitation is more difficult to estimate, but snowy conditions are common due to convective precipitation, as observed over Lewis Glacier [22].

Oblong tarn is nearly rectangular shaped with a long axis length of 180 m, a short axis of 90 m, and 1.6 ha surface area with a maximum depth of 10.7 m in August 2010 (Dirk Verschuren et al., Ghent University, unpublished data). Sediments deposited into the lake during the late Holocene were fine-grained minerogenic sediments from glacial meltwaters and till deposits, as well as authigenic and soil-derived organic matter. The proximity of the glacier to the tarn has fluctuated between 150 m in 1899 to 17 m in 1919, and it has since retreated considerably [45] indicating a rapid local response to global warming. Thus, the lake receives a large input of meltwater and fine inorganic sediments from the glacier and has done so throughout the Holocene. The sediment record of Oblong Tarn was collected on two excursions to the site and was previously studied to examine past glacial fluctuations by analyzing the first sediment core for x-ray density, sediment water content and organic/inorganic variations (loss-on-ignition) [44]. The second core was used to further examine glacier history [45] and oxygen isotopes derived from biogenic silica, primarily diatoms [46].

## Methods

### Field methods

Fieldwork permission, acknowledged in previous publications [44, 45], was granted by the Office of the Vice President of Kenya and local agreement by Kenya Wildlife Service at Mount Kenya National Park. The subsamples analysed for this study were not subject to further permissions. The authors affirm that the fieldwork did not involve endangered or protected species. In 1983, a raft built on two inflatable rubber boats was floated onto the lake and a wire-operated Livingstone piston corer (90 mm internal diameter) was used to collect a sediment core from 9.5 m water depth. The core was collected in two drives that overlapped to create a 245 cm stratigraphy, which did not reach the base of the soft sediments in the lake [44]. A second core was extracted in three overlapping drives in February 1986 using a 75 mm internal diameter Livingstone piston corer that reached the firm sediment base at ~550 cm [45]. An illustration of the core collection is presented in Supporting information S1 Fig. The two cores were independently radiocarbon dated and the temporal overlap of these cores was used to create a composite palynostratigraphy.

### Age-depth models

For the 1983 core, four conventional radiocarbon dates derived from bulk sediment were obtained from the Radioactive Dating Laboratory, Stockholm, Sweden. In the 1986 core, five conventional radiocarbon dates were collected from bulk sediments. These age determinations were used to build new age-depth models using BACON version 2.2 in the statistical programming language R version 3.0.2 [53–54]. Radiocarbon ages (^14^C year BP) were calibrated using the IntCal13 calibration curve [55]. Ages were interpolated using the weighted mean within a 95% confidence envelope of the densities of ~8 million Markov Chain Monte Carlo random walk iterations through the probability densities of the calibrated radiocarbon dates.

### Pollen analysis

Twenty eight subsamples of 0.5 cm^3^ of wet sediment were taken from 1-cm-thick levels spaced at 5 to 10 cm intervals down the 1983 core and twenty two subsamples were taken from the 1986 core. Pollen preparations followed standard sequential chemical digestions with HCl, KOH, HF, and acetolysis to remove organic, carbonate, and siliceous compounds and to prepare the pollen for microscopy, with rinses of deionized water, glacial acetic acid, or ethanol between digestions [56]. Tablets of *Eucalyptus* counter grains (batch 903722: 16,180 ± 1460 grains per table) were added to permit pollen concentration calculations for the 1983 core only [57]. Pollen grains were enumerated under an optical microscope at 400-1000x magnification and achieved terrestrial pollen grain counts of 520–1004 grains (mean = 630, σ = 122). Pollen identifications were aided by using a pollen reference collection at the Laboratoire de palynologie in Montpellier, France, a collection of photographs created at the laboratory, and published references [58–64]. Relative abundances were calculated from the total pollen sum. Pollen taxonomy was organized into plant functional groups for presentation using published pollen and vegetation assemblage records [63, 65–72]. Pollen data were analysed using C2 version 1.7.2 [73] and R [54] and plotted in C2. A principal components analysis of square-root transformed pollen percentages was done using C2 and the change function of the PaleoMAS package [74] was used for a rate of change (RoC) analysis of the dissimilarities between the pollen assemblage samples.

The sediment organic content were estimated by loss-on-ignition (LOI) analysis for the 1983 core and the values were digitized from the original published graph (from figure number 2 in [44]) using Data Thief III software [75].

## Results

Data for this study are available through the Harvard Dataverse data archive [76].

### Stratigraphy and age-depth model

**Fig 3:**
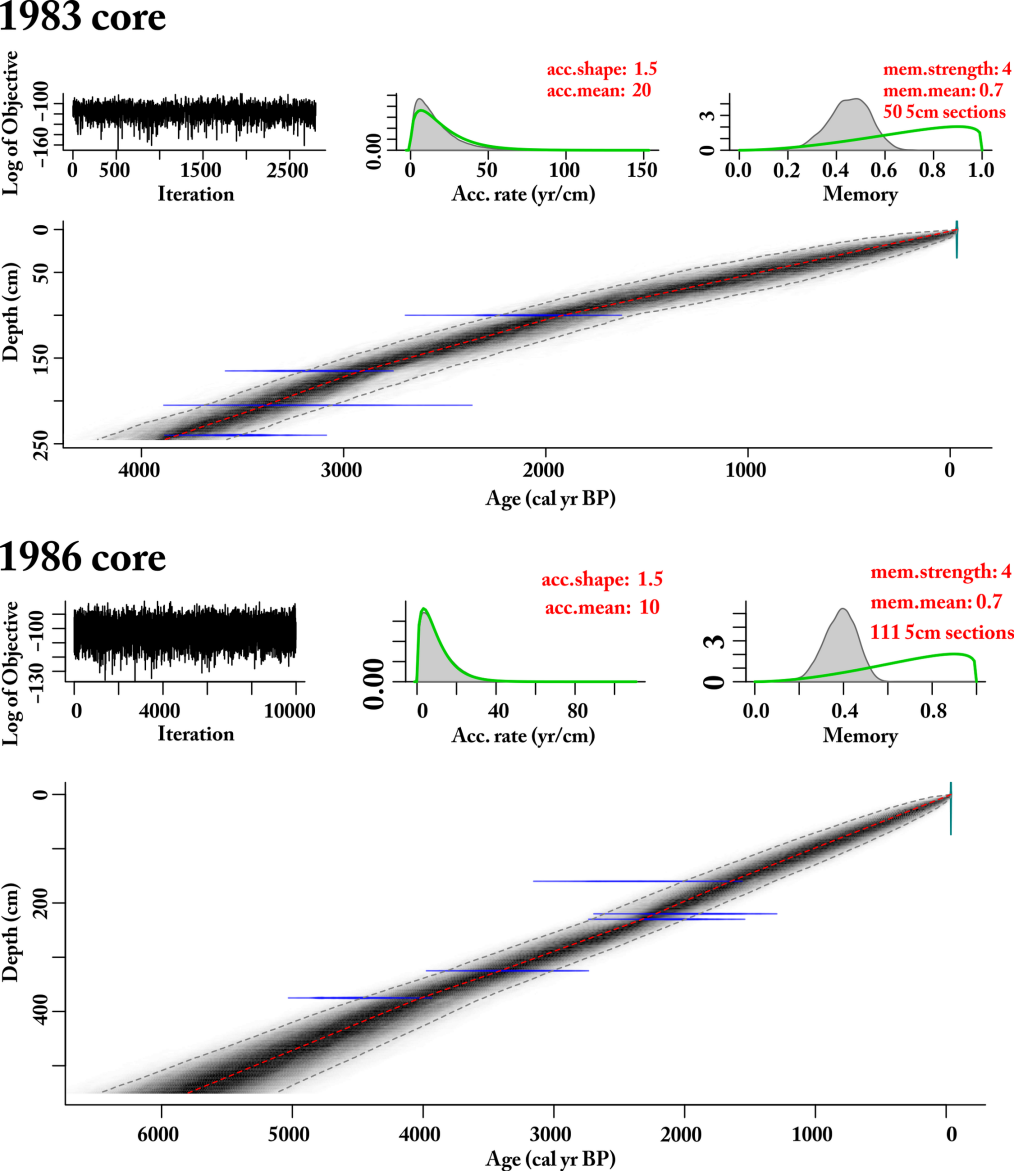
Radiocarbon dates and age-depth models for Oblong Tarn sediment cores. Age-depth models (red dotted line), random walks (greyscale), and 95% CI (dotted grey lines) of IntCal13 [55] calibrated radiocarbon dates (distributions in blue) with parameter settings (top right red font) for each of the Oblong Tarn sediment cores (Table 2; [53–54]). Ages reported as calibrated year BP (before present, 1950 CE). Pollen samples were taken from both the 1983 core [44] and the 1986 core [45] to create a composite stratigraphy.

Oblong Tarn sediments were diffusely banded, containing organic-rich (10–15%) and fine-grained glaciofluvial clastic-rich (<10% organic) sediments [44]. The loss-on-ignition (LOI) organic values ranged between 5–15% sediment dry weight and changes were dominantly influenced by variations in inorganic clastic input from glacial meltwater [44]. The age-depth model presented here (Fig 3) is comparable to both the original published model that used linear interpolation between the midpoint calibrated dates [44] and the independent age-depth model developed from the second core collected February 1986 [45]. This second coring expedition collected deeper soft sediments to a depth of 550 cm below the water-sediment interface; below this depth was either till or other hard sediments [45]. This new age-depth model included the radiocarbon date at 90–110 cm depth (St-9204) in the 1983 core that was rejected from the original age-depth model because that model relied on linear interpolation through the calibrated radiocarbon date midpoints (Table 2) [44, 77]. The BACON model made full use of the calibrated radiocarbon age probabilities and strongly suggested a continuous accumulation of sediments through time. The chronostratigraphic composite was built with the ages for each pollen sample using the separate age-depth model for each independent core to create the palynostratigraphy. Although the two cores were collected from the central area of the lake with slightly different sediment accumulation rates, the independent age-depth models permit compositing the samples.

**Table 2:** Radiocarbon dates from Oblong Tarn sediment core.

Reference	Depth (cm)	Radiocarbon age (^14^C yr)	Error 2σ (± yr)
1983 core (–33 cal yr BP)			
core top	0		
St-9204*	90–110	2115	140
St-9073	160–170	2995	135
St-9309	195–215	3000	275
St-9045	235–245	3275	135
core base	245		
1986 core (–36 cal yr BP)			
core top	0		
St-10647	150–160	2320	320
St-10648	210–230	1930	245
St-10863	220–240	2100	190
St-10682	310–340	3105	245
St-10683	360–390	4030	155
sediment base	~550		

Age determinations of bulk sediment samples used for conventional radiocarbon dating of the Oblong Tarn cores collected in 1983 [44] and 1986 [45].

*Date was not used in the original age-depth model [44]. Data are also available in the CARD database [76, 103].

### Pollen record

**Fig 4:**
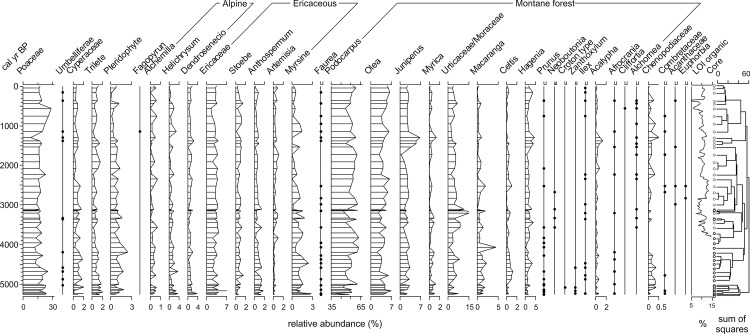
Pollen assemblages from the Oblong Tarn sediment cores. Pollen diagram of Oblong Tarn, Mount Kenya. Black circles show taxa with <1% relative abundances. LOI organic content (% dry sediment weight) estimated from the 1983 core [44]. At right, open black circles designate pollen samples from the 1983 core and open grey circles designate 1985 core subsamples. CONISS dendrogram shows that the entire record is contained within a single pollen assemblage zone with no statistically significant assemblage changes.

Pollen sampling intervals in the core averaged 133 years (range 47–200 years). Thirty six pollen taxa were identified in the record of which thirteen occurred with <1% relative abundance (Table 3, Fig 4). Pollen assemblage zones, defined by CONISS [78], were found to be not significant using a broken stick test [79] but a visible transition at 1500 cal yr BP occurred due to changes in the abundances of the dominant taxa (Fig 4). The pollen assemblage was predominantly derived from extra-local and regional pollen source areas.

**Table 3:** Pollen taxa observed in Oblong Tarn sediments.

Family	Pollen type [104]	Alp	Er	MF	LF	W-S	Wet	Group
Cyperaceae	Cyperaceae	x	x			x	X	all
	Trilete	x	x	x	x	x	x	all
	Pteridophyte	x	x	x	x	x	x	all
Poaceae	Poaceae	x	x	x	x	X	x	all
Polygonaceae	Polygonaceae	x	x	x	x	x	x	all
Umbelliferae	Umbelliferae	x	x	x	x	x		all
Rosaceae	*Alchemilla*	x						Alp
Asteraceae	*Helichrysum*	X	(x)	(x)	(x)	(x)		Alp
Asteraceae	*Dendrosenecio*-type	X	(x)					Alp
Asteraceae	*Stoebe*		x					Er
Asteraceae	*Artemisia*		X					Er
Ericaceae	Ericaceae		X	x				Er
Primulaceae	*Myrsine*		X	x				Er
Rosaceae	*Cliffortia*		X	x				Er
Rubiaceae	*Anthospermum*		X			x		Er
Aquifoliaceae	*Ilex*			X				MF
Cornaceae	*Afrocrania*			x	x			MF
Cupressaceae	*Juniperus*			X	x			MF
Euphorbiaceae	*Macaranga*			X				MF
Euphorbiaceae	*Neoboutonia*			x				MF
Euphorbiaceae	*Croton*			x	X			MF
Rutaceae	*Zanthoxylum*			x	x			MF
Euphorbiaceae	*Acalypha*			X	x	x		MF
Euphorbiaceae	*Alchornea*			X				MF
Myricaceae	*Myrica*			X				MF
Oleaceae	*Olea*			X				MF
Podocarpaceae	*Podocarpus*			X				MF
Rosaceae	*Hagenia*			X				MF
Rosaceae	*Prunus*			x	x			MF
Ulmaceae	*Celtis*			X	x			MF
Urticaceae/ Moraceae	Urticaceae/ Moraceae			X	x	x		MF
Proteaceae	*Faurea*				x	x		LF
Acanthaceae	Acanthaceae	x	x			X		W-S
Amaranthaceae/ Chenopodiaceae	Amaranthaceae/ Chenopodiaceae					X		W-S
Combretaceae	*Combretum*			X	x			W-S
Euphorbiaceae	*Euphorbia*				(x)	x		W-S

Pollen taxa assignments to biomes for vegetation interpretation through harmonizing published pollen records in the area [41, 63, 65–72, 92, 104]. Pollen groups are listed and the classification used in this study. Alp, alpine; Er, ericaceous; MF, montane forest; LF, lower forest; W-Sav, woody savannah; Wet, wetlands. Symbols: X, dominantly occurs; x, present; (x), occasionally present.

*Podocarpus* was consistently the most abundant taxon, varying between 47–62% and Poaceae relative abundances varied from 7–28%. The predominant montane forest pollen taxa, other than *Podocarpus*, were *Olea*, which varied between 3–6%, *Juniperus* (1–6%) and *Macaranga* (≤2%). Ericaceae abundances varied between 3–4% while all other taxa were below 2%. After 1500 cal yr BP, there was little change in *Podocarpus* and *Artemisia* pollen, and increased *Olea*, *Juniperus*, *Myrica*, Urticaceae, *Hagenia*, *Acalypha* and *Dendrosenecio*-type. *Myrsine*, *Macaranga* and pteridophyte-type pollen abundances decreased through the record. *Juniperus* and *Hagenia* both peaked centered between 1500–1100 cal yr BP, concomitant with slight increases in *Myrica*, *Acalpypha*, and Chenopdiaceae. Throughout the record, unidentified pollen averaged 5% (15–65 grains) of the total sum per sample.

**Fig 5:**
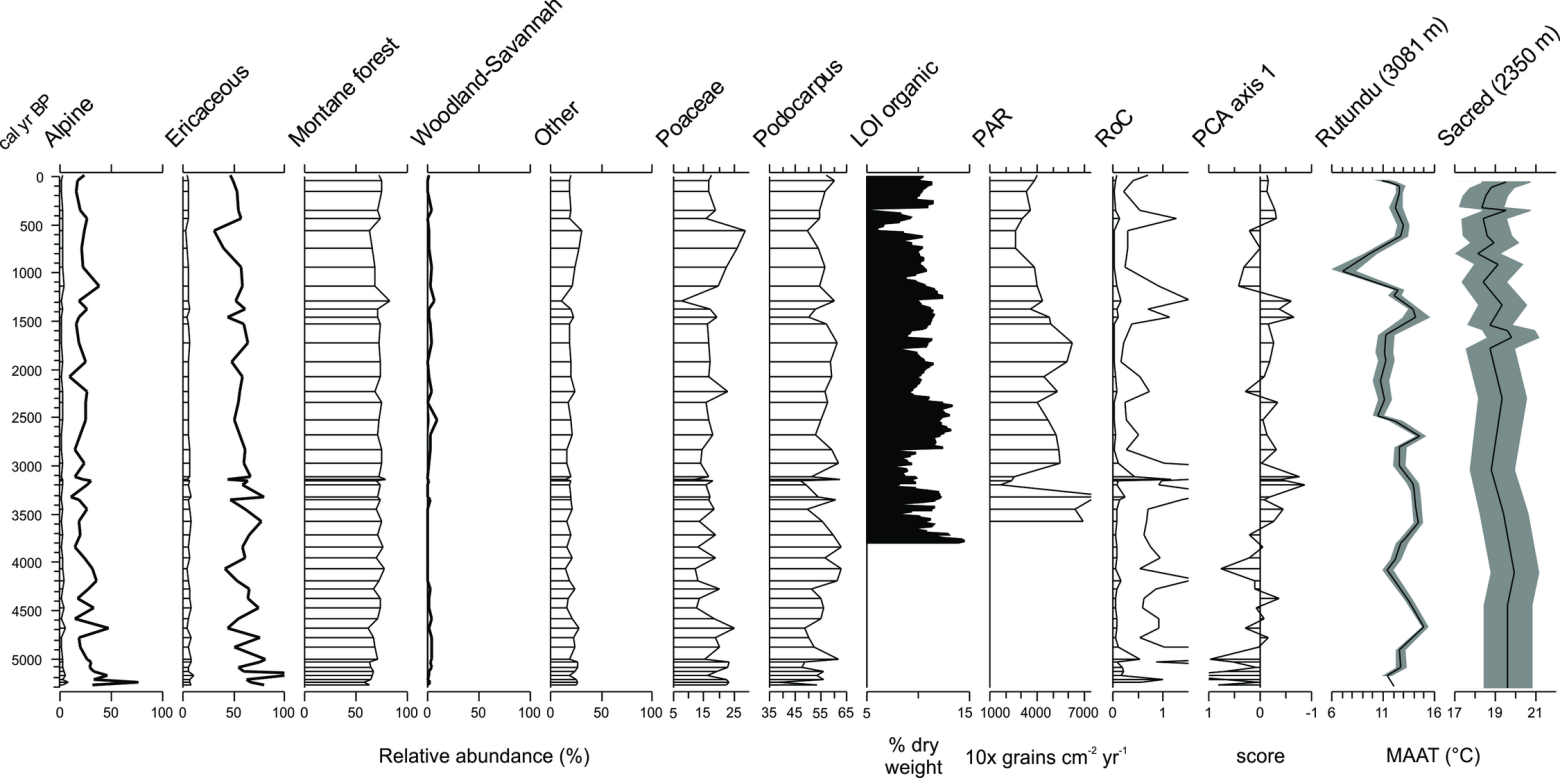
Pollen taxa groups and other palaeoenvironmental records for comparison. Taxonomic grouping of pollen relative abundances by biome. Alpine, ericaceous and woodland-savannah taxa groups shown with 10x exaggeration (dark black line). LOI and PAR derived from the 1983 core [44]. Rate of Change (RoC) analysis index values [74] and principal component axis one scores (19.5% variance explained) of square-root transformed pollen percentages. PCA scores are reversed for comparison with temperature reconstructions of mean annual air temperatures (MAAT) [103] from Rutundu Lake (in the lower ericaceous vegetation) [82] and Sacred Lake (within montane forest) [82, 105, 106] on Mount Kenya (elevation of the lake sites shown in m asl; shaded grey envelope shows 95% confidence interval).

Alpine taxa included *Alchemilla*, *Helichrysum*, and *Dendrosenecio*-type [63] and their abundance varied between 1–3%, while Ericaceae abundance varied from 4 to 8% (Fig 5). The dominant pollen were montane forest taxa that varied from 63–85% (14–24% excluding *Podocarpus*). Woodland and savannah taxa were <1%. The cosmopolitan taxa ranged from 10–30% of the total sum, primarily comprised of Poaceae. Total pollen accumulation rates (PAR) were calculated for the 1983 core and showed a general decrease in PAR from 3500 cal yr BP to present (Fig 5).

The first principal component explained 19.5% of the variance and scores showed little variability, with a tendency towards more negative values prior to 4100 cal yr BP and between 1000–500 cal yr BP (Fig 5). The rate of change analysis of the pollen assemblage shows a slight tendency for higher values from the beginning of the record until 3100 cal yr BP (mean = 0.11, excluding extreme values) and lower values from 3100 cal yr BP to present (mean = 0.06). Peak values were observed prior to 5000 and at 3100 cal yr BP (Fig 5).

## Discussion

Glacial advances in the Hausburg Valley occurred 6000–5000 years ago [8, 42] and Oblong Tarn likely formed after regression of the local glacier [42]. The basal age of the Oblong Tarn basin, dated to 5300 yr BP, fits well with the glacial history of Mount Kenya and the assemblage of mid-Holocene-aged morainal deposits [3, 5, 80]. The pollen assemblages of Oblong Tarn since 5300 cal yr BP were rather unresponsive to broad-scale environmental changes that have been documented for the Afrotropical region [81]. The apparent low degree of variation limits a comparison with the lacustrine inorganic sediment influx from the glacier to infer local-scale relationships between environmental and alpine vegetation changes [44–45]. The slight variability in the overall pollen assemblages expressed as PCA axis 1 scores, does show a negative relationship to temperatures reconstructed from a mid-elevations site (Rutundu Lake [82], Fig 5) even given the centennial-scale uncertainties in the age-depth, and this may reflect the effects of temperature changes on montane forest composition. The long term decrease in relative abundances of alpine and ericaceous pollen since the mid-Holocene may reflect decreased pollen production by the cold ecosystem taxa due to late Holocene cooling at upper elevations [82]. There was also no conspicuous evidence of significant anthropogenic environmental modifications, notably taxa associated with disturbance from deforestation [72] around the mountain and on the lower elevations of Mount Kenya. It is difficult to disentangle the climatic, fire regime variability, and anthropogenic contributions to the *Podocarpus* pollen record; although changes to all of these mechanisms could have influenced or varied in relative importance since the mid-Holocene.

The upwind transport to the site and channeling of winds at the top of the mountain [83] homogenizes the pollen from a large area of the windward mountain, spanning multiple vegetation zones [84–86]. The record may be further confounded by secondary pollen input through meltwaters from the ablation of César and Josef Glacier at the head of valley causing some temporal aggregation in the pollen signal; although the influx of minerogenic material seems to dilute pollen accumulation rates (PAR, Fig 5). Pollen transported from long distances has been observed in snow and ice and taphonomic studies would be useful for quantifying secondary pollen influxes and estimating temporal aggregation or lag effects in pollen records from proglacial lake sediment. The high abundance of montane forest taxa and low abundances of alpine and ericaceous taxa could be due to the low pollen production of higher elevation plants and the large source area of the montane taxa, and other factors such as changes in transportation of aerophilous pollen types [87–88]. An interesting feature is illustrated by the increased pollen abundances of *Juniperus*, *Hagenica*, *Myrica*, *Acalypha* motane forest taxa at 1500–1100 cal yr BP, as an example of minor short-termed vegetation changes that span multiple samples. This centennial-scale minor increase in these taxa occurred during a period of increased reconstructed temperatures at Lake Rutundu (Fig 5). These results indicate a need for modern pollen-vegetation cover studies in upper elevations.

### Oblong Tarn pollen record

The most frequent alpine pollen taxa were *Alchemilla*, *Helichrysum* and *Dendrosenecio*-type, of which many species are endemic to Mount Kenya and neighbouring alpine elevations [65]. The abundance of pollen from alpine taxa was relatively constant over the past 3500 years, although *Dendrosenecio*-type pollen were found in higher abundance before 5000 cal yr BP and from 1500–1300 cal yr BP, potentially indicative of local glacial retreat and soil stabilization [28] following the mid-Holocene glacial maximum. This occurred during a period of a prolonged (1500–550 cal yr BP) reduction in glacier activity on Mount Kenya, with higher organic content (10–12%) that generally decreased to a seemingly abrupt onset of minerogenic sediments at 550 cal yr BP [44]. High-resolution loss-on-ignition analysis of the uppermost sediments showed that organic content was <10% from 500–150 cal yr BP and increased from 3–18% from 150 to present (Figs 4 and 5).

When interpreted by aggregating the pollen taxa into pollen taxa groups (Fig 5), changes in the record were more apparent, including an overall decreasing trend in alpine (3–1.5%) and ericaceous taxa (8 to 4%) and an early increase, followed by a sustained high abundance of *Podocarpus* pollen. Montane forest pollen percentages increased between 5000–4000 cal yr BP, then leveled with little variation until they decreased by 1000–500 cal yr BP, and finally again increased between 500 cal yr BP to present. Woodland and savannah taxa were very low throughout the record and were absent from 4200–3000 cal yr BP. It is difficult to link to discrete mechanisms, but these variations could be related to climatic influences on low- and mid-elevation vegetation cover or wind circulation across the mountain. Generally, rates of change are slightly higher prior to 3100 cal yr BP and lower subsequently. This decrease is associated with a stabilization of the percentages of pollen taxa from the alpine and ericaceous zones at this time. The RoC analysis assumes linear accumulation rates, thus, peaks in the RoC values could be related to high-frequency variability in sedimentationthat is not resolved by the age-depth model. For example, the peak around 3100 cal yr BP is associated with some variation in LOI values Temperature reconstructions from Mount Kenya suggest that temperatures have generally decreased from 5000 cal yr BP to recent, but temperatures at higher elevations decreased much more and had much higher multi-centennial variability [82].

From 1100–400 cal yr BP, *Podocarpus* abundance decreased and Poaceae increased, which could be due to several processes, including an increase in local grasses, decreased *Podocarpus* abundance in the montane forests, or changes in the dominant wind direction carrying pollen from the bamboo zone or montane forest. *Podocarpus* is likely overrepresented due to the high production and long-distance transport from lower on the mountain [35, 89]. Other taxa that are potentially overrepresented in pollen records relative to surrounding vegetation include Ericaceae, Rosaceae (*Hagenia*), Euphorbiaceae (*Macaranga*) and Araliaceae [71, 89].

### Comparison with late Holocene records on Mount Kenya

One of the more conspicuous aspects of the Oblong Tarn pollen record is the high relative abundances of Poaceae and *Podocarpus*. A late Holocene increase in *Podocarpus* abundances has been found in multiple pollen records from Mount Kenya, including Hobley Valley mire [42]; Rumuiku Swamp [41]; Sacred Lake [61]; Lake Rutundu [39] and Lake Nkunga at the base of mountain [90]. Similarly, a sediment record from Hobley Valley mire contained high relative abundances of Poacaeae (10–50%) and *Podocarpus* pollen during the past ~3270 years BP [42]. This high elevation mire also contained similar abundances as the Oblong Tarn record for *Alchemilla*, *Helichrysum*, *Dendrosenecio* and taxa from the ericaceous zone. At Rumuiku Swamp (2150 m asl), *Podocarpus* relative abundances were very low at the beginning of the Holocene, but increased to 20% by the late Holocene [41]. Oxygen isotope values derived from diatom frustules in the sediments on Small Hall and Simba Tarns both abruptly increased at 3000 yr BP suggesting drier conditions than previously, and the values at Hausberg Tarn increased around 2500 cal yr BP [47]. This association between hydroclimate and vegetation change in the Oblong Tarn pollen record suggest an important link between moisture regimes and vegetation at mid to high elevations at centennial and longer timescales.

### Comparison to other East African mountain records

The pollen record from Oblong Tarn is similar in composition and abundances to many late Holocene pollen diagrams from high elevation sites in equatorial east Africa. Pollen records from Lake Kimilili, Mount Elgon (from 4150 m asl, [63]) and as far as Lake Mahoma, Ruwenzori Mountains, Uganda [91] all show evidence of increased *Podocarpus* in the late Holocene. Alpine, ericaceous and montane forest taxa dominated the Lake Kimilili record. This record showed the dominance of Poaceae and *Podocarpus* (20–50%) over the past 4000 years, with Poaceae decreasing and *Podocarpus* increasing since the beginning of the Holocene. The Oblong Tarn record is also similar to late Holocene records from Koitoboss Bog (3940 m asl) and Laboot Swamp (2880 m asl), Mount Elgon, and to an undated, high-elevation pollen record from Scout Hut Mire (3380 m asl), Mount Elgon [63]. The pollen record from a soil pit WeruWeru26 (2650 m asl), on the southern slopes of Kilimanjaro, is located in the montane forest vegetation zone but also contained abundant Poaceae (10%) and high *Podocarpus* (10–40%) abundances during the late Holocene [92].

Abundant Poacaeae and *Podocarpus* is a common feature to high elevation pollen records in the region unless there are ericaceous and montane forest taxa locally to increase the pollen representation from these taxa. The abundant Poaceae and *Podocarpus* pollen at Oblong Tarn may be reinforced by secondary deposition of these grains from the melting seasonal snow and glacier into the lake, resulting in dilution of the original wind-deposited pollen. Other high elevation records, such as Lake Kimilili, in a non-glaciated catchment do show significant variation in taxa during the mid-Holocene but variability is dampened during the late Holocene rise in *Podocarpus* [63, 91]. Sites such as Koitoboss Bog show stronger variability in montane forest and Afroalpine taxa before and following the *Podocarpus* increase [63].

Modern pollen deposition in eastern Africa has shown the signature of vegetation associations along altitudinal gradients [71, 93–94] and climate [95–96]. Additional high- and mid- elevation records and modern pollen rain calibration studies [71, 97] are necessary to further examine the impact of *Podocarpus* expansion on montane forest ecology and representivity in the available pollen sites across equatorial eastern Africa.

## Conclusions

The Oblong Tarn pollen record consists primarily of montane forest and ericaceous pollen transported from lower elevations. The late Holocene record is dominated by Poaceae and *Podocarpus* grains, in agreement with previously published records from Mount Kenya and neighbouring high-elevation sites. The pollen assemblages showed little variation over the late Holocene with statistically insignificant changes in abundance variations prior to 4000 cal yr BP and between 1500–1300 cal yr BP during a prolonged period of glacial inactivity in the valley. These minor changes could relate to climatic variability and suggest some sensitivity in the record to montane forest vegetation changes. When aggregated into functional groupings, changes are seen in the pollen record that can be interpreted in the context of other studies in the region. In particular, a long-term cooling over the past 5000 years is seen in slight decreases in pollen of local taxa and increases in pollen transport from lower elevations that stabilize by 3100 cal yr BP to present.

## Supporting information

S1 Fig Sediment core collection from Oblong Tarn, Mount Kenya. Sediment cores recovered during two fieldwork expeditions in 1983 and 1986.
(PDF)

## References

[1] Mahaney WC. Holocene glaciations and paleoclimate of Mount Kenya and other East African mountains. Quaternary Science Reviews. 1988 1 1;7(2):211–25.

[2] Mahaney WC, Barendregt RW, Vortisch W. Quaternary glaciations and palaeoclimate of Mount Kenya, East Africa In Glacier fluctuations and climatic change. Dordrecht: Springer Netherlands, 1989 pp. 13–35.

[3] Harmsen R, Spence JR, Mahaney WC. Glacial interglacial cycles and development of the Afroalpine ecosystem on East African Mountains II. Origins and development of the biotic component. Journal of African Earth Sciences (and the Middle East). 1991 1 1;12(3):513–23.

[4] Baker BH. Geology of the Mount Kenya area. Nairobi: Geological Survey of Kenya, Ministry of Natural Resources; 1967.

[5] Johansson L, Holmgren K. Dating of a moraine on Mount Kenya. Geografiska Annaler. Series A. Physical Geography. 1985 1 1:123–8.

[6] Young JAT, Hastenrath SL. Glaciers of Africa (G-3); in Williams, R.S., Jr., and Ferrigno, J.G., eds., Satellite image atlas of glaciers of the world: U.S. Geological Survey Professional Paper 1386-G (Glaciers of the Middle East and Africa), 1991.

[7] MacKinder JH. Mount Kenya in 1899. Geographical Journal. 1930; 76: 529–534.

[8] Karlen W, Fastook JL, Holmgren K, Malmström M, Matthews JA, Odada E, et al Glacier Fluctations on Mount Kenya since-6000 Cal. Years BP: Implications for Holocene Climatic Change in Africa. Ambio-Journal of Human Environment Research and Management. 1999 8 1;28(5):409–18.

[9] Hastenrath S. Glaciological studies on Mount Kenya 1971–2005. Madison: University of Wisconsin, 2005.

[10] Hastenrath S. Recession of equatorial glaciers: a photo documentation Madison: Sundog Publishing, 2008.

[11] Rosqvist G. Quaternary glaciations in Africa. Quaternary Science Reviews. 1990;9:281–297.

[12] Mutai CC, Ward MN. East African rainfall and the tropical circulation/convection on intraseasonal to interannual timescales. Journal of Climate. 2000 11;13(22):3915–39.

[13] Marchant R, Mumbi C, Behera S, Yamagata T. The Indian Ocean dipole–the unsung driver of climatic variability in East Africa. African Journal of Ecology. 2007 3 1;45(1):4–16.

[14] Hastenrath S. Net balance, surface lowering, and ice-flow pattern in the interior of Lewis Glacier, Mount Kenya, Kenya. Journal of Glaciology. 1983 1 1;29(103):392–402.

[15] Hastenrath S. The glaciers of equatorial East Africa Dordrecht/Boston/Lancaster: D. Reidel Publishing Company, 1984.

[16] Osmaston HA, Harrison SP. The Late Quaternary glaciation of Africa: A regional synthesis. Quaternary International. 2005;138:32–54.

[17] Carrivick JL, Tweed FS. Proglacial lakes: character, behaviour and geological importance. Quaternary Science Reviews. 2013 10 15;78:34–52.

[18] Wiesmann U, Gichuki FN, Kiteme BP, Liniger H. Mitigating conflicts over scarce water resources in the highland-lowland system of Mount Kenya. Mountain Research and Development. 2000 2;20(1):10–5.

[19] Aeschbacher J, Liniger H, Weingartner R. River water shortage in a highland–lowland system: a case study of the impacts of water abstraction in the Mount Kenya region. Mountain Research and Development. 2005 5;25(2):155–62.

[20] Notter B, MacMillan L, Viviroli D, Weingartner R, Liniger HP. Impacts of environmental change on water resources in the Mt. Kenya region. Journal of Hydrology. 2007 9 20;343(3):266–78.

[21] Mölg T, Cullen NJ, Hardy DR, Kaser G, Nicholson L, Prinz R, et al East African glacier loss and climate change: Corrections to the UNEP article “Africa without ice and snow”. Environmental Development 2013;6:1–6.

[22] Nicholson LI, Prinz R, Mölg T, Kaser G. Micrometeorological conditions and surface mass and energy fluxes on Lewis Glacier, Mt Kenya, in relation to other tropical glaciers. The Cryosphere 2013;7(4):1205–1225.

[23] Mahaney WC, Spence JR. Lichenometry of Neoglacial moraines in Lewis and Tyndall cirques on Mount Kenya. Zeitshrift fur Gletscherkunde und Glazialgeologie. 1989; 25: 175–186.

[24] Mizuno K. Succession processes of alpine vegetation in response to glacial fluctuations of Tyndall Glacier, Mt. Kenya, Kenya. Arctic and Alpine Research. 1998 11 1:340–8.

[25] Mizuno K. Glacial fluctuation and vegetation succession on Tyndall glacier, Mt Kenya. Mountain Research and Development. 2005 2;25(1):68–75.

[26] Mizuno K. Vegetation succession in relation to glacial fluctuation in the high mountains of Africa. African Study Monographs. 2005; Supplementary Issue 30: 195–212.

[27] Mahaney WC, Boyer MG. Late glacial soil catena in upper Teleki Valley, Mount Kenya Afroalpine area. Journal of African Earth Sciences (1983). 1987 1 1;6(5):731–40.

[28] Mizuno K, Fujita T. Vegetation succession on Mt. Kenya in relation to glacial fluctuation and global warming. Journal of vegetation science. 2014 3 1;25(2):559–70.

[29] Billings WD, Mooney HA. The ecology of arctic and alpine plants. Biological reviews. 1968 11 1;43(4):481–529.

[30] Hemp A. Introduced plants on Kilimanjaro: tourism and its impact. Plant Ecology. 2008 7 1;197(1):17–29.

[31] Fries RE, Fries TCE. Phytogeographical researches on Mt. Kenya and Mt. Aberdare, British East Africa. Kungl. Svenska vetenskapsakademiens handlingar, Tbedje Serien. Stockholm: Band 25. No 5. Almqvist & Wiksellr Boktryckeri-a.-b. 1948.

[32] Knox EB, Palmer JD. Chloroplast DNA variation and the recent radiation of the giant senecios (Asteraceae) on the tall mountains of eastern Africa. Proceedings of the National Academy of Sciences. 1995 10 24;92(22):10349–53. 10.1073/pnas.92.22.10349 PMC40794 7479782

[33] Steinbauer MJ, Field R, Grytnes JA, Trigas P, Ah‐Peng C, Attorre F, et al Topography‐driven isolation, speciation and a global increase of endemism with elevation. Global Ecology and Biogeography. 2016 9 1;25(9):1097–107.

[34] Spence JR. Plant succession on glacial deposits of Mount Kenya, East Africa In (Mahaney W.C., ed.) Quaternary and Environmental Research on East African Mountains. Rotterdam: Balkema, 1989 pp. 279–290.

[35] Coetzee JA. Evidence for a considerable depression of the vegetation belts during the Upper Pleistocene on the East African mountains. Nature. 1964 11 7;204(4958):564–6.

[36] Ficken KJ, Street-Perrott FA, Perrott RA, Swain DL, Olago DO, Eglinton G. Glacial/interglacial variations in carbon cycling revealed by molecular and isotope stratigraphy of Lake Nkunga, Mt. Kenya, East Africa. Organic Geochemistry. 1998 11 30;29(5):1701–19.

[37] Huang Y, Street-Perrott FA, Perrott RA, Metzger P, Eglinton G. Glacial–interglacial environmental changes inferred from molecular and compound-specific δ 13 C analyses of sediments from Sacred Lake, Mt. Kenya. Geochimica et Cosmochimica Acta. 1999 5 31;63(9):1383–404.

[38] Olago DD, Street-Perrott FA, Perrott RA, Odada EO. Late Holocene sedimentology and palaeoenvironment of Kiluli Swamp, Mount Kenya. African Journal of Science and Technology. 2003;4(2):12–23.

[39] Wooller MJ, Swain DL, Ficken KJ, Agnew AD, Street‐Perrott FA, Eglinton G. Late Quaternary vegetation changes around Lake Rutundu, Mount Kenya, East Africa: evidence from grass cuticles, pollen and stable carbon isotopes. Journal of Quaternary Science. 2003 1 1;18(1):3–15.

[40] Street-Perrott FA, Barker PA, Swain DL, Ficken KJ, Wooller MJ, Olago DO, et al Late Quaternary changes in ecosystems and carbon cycling on Mt. Kenya, East Africa: a landscape-ecological perspective based on multi-proxy lake-sediment influxes. Quaternary Science Reviews. 2007 7 31;26(13):1838–60.

[41] Rucina SM, Muiruri VM, Kinyanjui RN, McGuiness K, Marchant R. Late Quaternary vegetation and fire dynamics on Mount Kenya. Palaeogeography, Palaeoclimatology, Palaeoecology. 2009 12 1;283(1):1–4.

[42] Perrott RA. A high altitude pollen diagram from Mount Kenya: its implications for the history of glaciation. Palaeoecology of Africa and the Surrounding Islands. 1982;14:77–83.

[43] Niemelä T. Pellikka P4. Zonation and characteristics of the vegetation of Mt. Kenya. Taita Hills and Kenya, 2004 –seminar, reports and journal of a field excursion to Kenya. Expedition reports of the Department of Geography, University of Helsinki 2004; 40, 14–20.

[44] Karlén W. Glacier and climate fluctuations on Mount Kenya, East Africa. Zeitschrift fur Gletscherkunde und Glazialgeologie. 1985; 21: 195–201.

[45] Karlén W, Rosqvist G. Glacier fluctuations recorded in lacustrine sediments on Mount Kenya. National Geographic Research. 1988 3 1;4(2):219–32.

[46] Rietti-Shati M, Yam R, Karlen W, Shemesh A. Stable isotope composition of tropical high-altitude fresh-waters on Mt. Kenya, Equatorial East Africa. Chemical Geology. 2000 5 22;166(3):341–50.

[47] Barker PA, Street-Perrott FA, Leng MJ, Greenwood PB, Swain DL, Perrott RA, et al A 14,000-year oxygen isotope record from diatom silica in two alpine lakes on Mt. Kenya. Science. 2001 6 22;292(5525):2307–10. doi: 10.1126/science.1059612 11423656 10.1126/science.1059612

[48] Street-Perrott FA, Ficken KJ, Huang Y, Eglinton G. Late Quaternary changes in carbon cycling on Mt. Kenya, East Africa: an overview of the δ 13 C record in lacustrine organic matter. Quaternary Science Reviews. 2004 4 30;23(7):861–79.

[49] Mahaney WC. Ice on the equator: Quaternary geology of Mount Kenya Sister Bay, WI, US: Wm Caxton Ltd, 1990.

[50] Bhatt N. The geology of Mount Kenya In: Allen I. (ed.) Guide to Mount Kenya and Kilimanjaro. Nairobi: The Mountain Club of Kenya, 1991 pp. 54–66.

[51] Axelrod DI, Raven PH. Late Cretaceous and Tertiary vegetation history of Africa. In: Biogeography and Ecology of Southern Africa (Ed. M. J. A. Werger, W. Junk). The Hague, 1978. Pp. 77–130.

[52] Hastenrath S. 1985 Climate and circulation of the tropics D. Reidel Publishing Company.

[53] Blaauw M, Christen JA. Flexible paleoclimate age-depth models using an autoregressive gamma process. Bayesian analysis. 2011;6(3):457–74.

[54] R Development Core Team. R: A language and environment for statistical computing R Foundation for statistical computing 2015.

[55] Reimer PJ, Bard E, Bayliss A, Beck JW, Blackwell PG, Bronk Ramsey C, et al IntCal13 and Marine13 radiocarbon age calibration curves 0–50,000 years cal BP. Radiocarbon. 2013; 55(4):1869–1887.

[56] Fægri K, Iversen J. Textbook of Pollen Analysis. New York: Hafner Publishing Company, 1964.

[57] Benninghoff WS. Calculation of pollen and spores density in sediments by addition of exotic pollen in known quantities. Pollen et Spores. 1962; 4: 332–333.

[58] Erdtman G. Pollen morphology and plant taxonomy Angiosperms. Stockholm: Almqvist and Wiksell, 1952.

[59] Bonnefille R. Atlas des pollens d'Ethiopie. Principales espèces des forêts de montagne. Pollen Spores. 1971;13(1):15–68.

[60] Bonnefille R and Riollet G. 1980 Pollens des savanes d’Afrique orientale Paris: Editions du Centre National de la Recherche Scientifique.

[61] Coetzee JA. Pollen analytical studies in East and Southern Africa. Palaeoecology of Africa. 1967; 3: 1–146.

[62] Hamilton AC. Identification of east African Urticales pollen. Pollen Spores. 1976;18(1):27–66.

[63] Hamilton AC. Environmental history of East Africa–A study of the Quaternary. London: London Academic Press, 1982.

[64] Scott L. Late Quaternary fossil pollen grains from the Transvaal, South Africa. Review of Palaeobotany and Palynology. 1982 4 1;36(3–4):241–78.

[65] Coe M. The Ecology of the Alpine Zone of Mount Kenya. The Hague: Dr. W. Junk Publishers, 1967.

[66] Olago DO. Vegetation changes over palaeo-time scales in Africa. Climate Research. 2001 8 15;17(2):105–21.

[67] Rucina S. Kenya ecosystem dynamics: perspectives from high and low altitude ecosystems. York, UK: Unpublished Phd. University of York, 2011.

[68] Rucina SM, Muiruri VM, Downton L, Marchant R. Late-Holocene savanna dynamics in the Amboseli Basin, Kenya. The Holocene. 2010 8;20(5):667–77.

[69] Schüler L, Hemp A, Zech W, Behling H. Vegetation, climate and fire-dynamics in East Africa inferred from the Maundi crater pollen record from Mt Kilimanjaro during the last glacial–interglacial cycle. Quaternary Science Reviews. 2012 4 16;39:1–3.

[70] Schüler L. Studies on late Quaternary environmental dynamics (vegetation, biodiversity, climate, soils, fire and human impact) on Mt Kilimanjaro. Goettingen, Germany: Unpublished PhD. University of Goettingen, 2013.

[71] Schüler L, Hemp A, Behling H. Relationship between vegetation and modern pollen-rain along an elevational gradient on Kilimanjaro, Tanzania. The Holocene. 2014 6;24(6):702–13.

[72] Finch J, Marchant R, Courtney Mustaphi CJ. Ecosystem change in the South Pare Mountain bloc, Eastern Arc Mountains of Tanzania. The Holocene. 2017 doi: 10.1177/0959683616675937

[73] Juggins, S. 2007. C2: software for ecological and palaeoecological data analysis and visualization. https://www.staff.ncl.ac.uk/stephen.juggins/software/C2Home.htm

[74] Urrego D, Bush MB, Silman M, Correa-Metrio A, Ledru M, Mayle F, et al 2009 Millenial-scale ecological changes in tropical South America since the Last Glacial Maximum In: Vimieux F., Sylvestre F., and Khodri M., editors. Past climate variability from the Last Glacial Maximum to the Holocene in South America and surrounding regions. Springer, Paris.

[75] Tummers B. 2006. DataThief III software. Available: http://datathief.org/

[76] Courtney Mustaphi C, Gajewski K, Marchant R, Rosqvist G. Radiocarbon dates, sedimentology, and pollen counts from the late Holocene sediments of Oblong Tarn, Mount Kenya., Harvard Dataverse, V1. 2017. doi: 10.7910/DVN/BBDYUZ

[77] Telford RJ, Heegaard E, Birks HJB. The intercept is a poor estimate of a calibrated radiocarbon age. The Holocene. 2004; 14(2):296–298.

[78] Grimm EC. CONISS: a FORTRAN 77 program for stratigraphically constrained cluster analysis by the method of incremental sum of squares. Computers & Geosciences. 1987 1 1;13(1):13–35.

[79] Bennett KD. Determination of the number of zones in a biostratigraphical sequence. New Phytologist. 1996 1 1;132(1):155–70. 10.1111/j.1469-8137.1996.tb04521.x 33863055

[80] Mahaney WC. Reinterpretation of moraines at ∼4000 m in the Mount Kenya Afroalpine area. Palaeogeography, palaeoclimatology, palaeoecology. 1987 1 1;60:47–57.

[81] Marchant R, Hooghiemstra H. Rapid environmental change in African and South American tropics around 4000 years before present: a review. Earth-Science Reviews. 2004 8 31;66(3):217–60.

[82] Loomis SE, Russell JM, Verschuren D, Morrill C, De Cort G, Damsté JSS, et al The tropical lapse rate steepened during the Last Glacial Maximum. Science advances. 2017;3(1):e1600815 doi: 10.1126/sciadv.1600815 28138544 10.1126/sciadv.1600815 PMC5271593

[83] Solomon AM, Silkworth AB. Spatial patterns of atmospheric pollen transport in a montane region. Quaternary Research. 1986 3 1;25(2):150–62.

[84] Hedberg O. Vegetation belts of the East African mountains. Svensk Botanisk Tidskrift. 1951;45:140–202.

[85] Hemp A. Continuum or zonation? Altitudinal gradients in the forest vegetation of Mt. Kilimanjaro. Plant Ecology. 2006 5 1;184(1):27–42.

[86] Bussmann RW. Succession and regeneration patterns of East African mountain forests. A review. Systematics and Geography of plants. 2001 1 1:959–74.

[87] Markgraf V. Pollen dispersal in a mountain area. Grana. 1980 8 1;19(2):127–46.

[88] Swain DL. Late Quaternary palaeoecology of Mount Kenya, East Africa: investigating the potential impact of sub-ambient CO2 concentration on the distribution of montane vegetation. Swansea: Ph.D. Thesis, University of Wales, 1999.

[89] Marchant R, Taylor D. Pollen representivity of montane forest taxa in southwest Uganda. New Phytologist. 2000 6 1;146(3):515–25.

[90] Olago DO, Street-Perrott FA, Perrott RA, Ivanovich M, Harkness DD. Late Quaternary glacial-interglacial cycle of climatic and environmental change on Mount Kenya, Kenya. Journal of African Earth Sciences. 1999 10 1;29(3):593–618.

[91] Livingstone DA. Postglacial vegetation of the Ruwenzori Mountains in equatorial Africa. Ecological Monographs. 1967 2 1;37(1):25–52.

[92] Schüler L, Hemp A. Atlas of pollen and spores and their parent taxa of Mt Kilimanjaro and tropical East Africa. Quaternary International. 2016 12 15;425:301–86.

[93] Bonnefille R, Buchet G, Friis IB, Kelbessa E, Mohammed MU. Modern pollen rain on an altitudinal range of forests and woodlands in South West Ethiopia. Opera Botanica. 1993;121:71–84.

[94] Vincens A, Ssemmanda I, Roux M, Jolly D. Study of the modern pollen rain in Western Uganda with a numerical approach. Review of Palaeobotany and Palynology. 1997 3 1;96(1–2):145–68.

[95] Gajewski K, Lézine AM, Vincens A, Delestan A, Sawada M. Modern climate–vegetation–pollen relations in Africa and adjacent areas. Quaternary Science Reviews. 2002; 21(14), pp.1611–1631.

[96] Watrin J, Lézine AM, Gajewski K, Vincens A. Pollen–plant–climate relationships in sub-Saharan Africa. Journal of Biogeography. 2007 3 1;34(3):489–99.

[97] Courtney Mustaphi CJ, Githumbi E, Mutua J, Muriuki RM, Rucina SM, Marchant R. Ongoing sedimentological and palaeoecological investigations at Nyabuiyabui wetland, Kiptunga Forest Block, Eastern Mau Forest, Nakuru District, Kenya. Report to the Mau Forest Conservation Office, Kenya Forest Service, and the National Museums of Kenya Palaeobotany and Palynology Section. REAL contribution 002. 4 May 2014.

[98] Barker P, Street-Perrott FA, Perrott RA, Ficken K. Diatom productivity from a high altitude tropical lake on Mt. Kenya: preliminary results from Lake Rutundu. In: Proceedings, Societas Internationalis Limnologiae Conference, Dublin. Mitteilungen der Internationale Vereinigung fur Limnologie 27; 2000. Pp. 1003–1007.

[99] Mahaney WC, Boyer MG. Late glacial soil catena in upper Teleki Valley, Mount Kenya Afroalpine area. Journal of African Earth Sciences (1983). 1987 1 1;6(5):731–40.

[100] Ficken KJ, Wooller MJ, Swain DL, Street-Perrott FA, Eglinton G. Reconstruction of a subalpine grass-dominated ecosystem, Lake Rutundu, Mount Kenya: a novel multi-proxy approach. Palaeogeography, Palaeoclimatology, Palaeoecology. 2002 1 5;177(1):137–49.

[101] Barão L, De Cort G, Meire P, Verschuren D, Struyf E. Biogenic Si analysis in volcanically imprinted lacustrine systems: the case of Lake Rutundu (Mt. Kenya). Biogeochemistry. 2015 9 1;125(2):243–59.

[102] Konecky B, Russell J, Huang Y, Vuille M, Cohen L, Street-Perrott FA. Impact of monsoons, temperature, and CO_2_ on the rainfall and ecosystems of Mt. Kenya during the Common Era. Palaeogeography, Palaeoclimatology, Palaeoecology. 2014 2 15;396:17–25.

[103] Courtney Mustaphi C, Marchant R. A database of radiocarbon dates for palaeoenvironmental research in Eastern Africa. Open Quaternary. 2016; 2(3): 1–7.

[104] Vincens A, Lézine AM, Buchet G, Lewden D, Le Thomas A. African pollen database inventory of tree and shrub pollen types. Review of Palaeobotany and Palynology. 2007 6 30;145(1):135–41.

[105] Naafs BDA, Inglis GN, Zheng Y, Amesbury MJ, Biester H, Bindler R, et al 2017 Introducing global peat-specific temperature and pH calibrations based on brGDGT bacterial lipids. Geochimica et Cosmochimica Acta.

[106] Loomis SE, Russell JM, Ladd B, Street-Perrott FA, Damsté JSS. 2012 Calibration and application of the branched GDGT temperature proxy on East African lake sediments. Earth and Planetary Science Letters, 357, pp.277–288.

