# Supplementary material for: A late Holocene pollen record from proglacial Oblong Tarn, Mount Kenya
Source: PLoS One. 2017 Sep 19;12(9):e0184925. doi: 10.1371/journal.pone.0184925 (PMC5604990; doi:10.1371/journal.pone.0184925)

Core collection from the center of Oblong Tarn

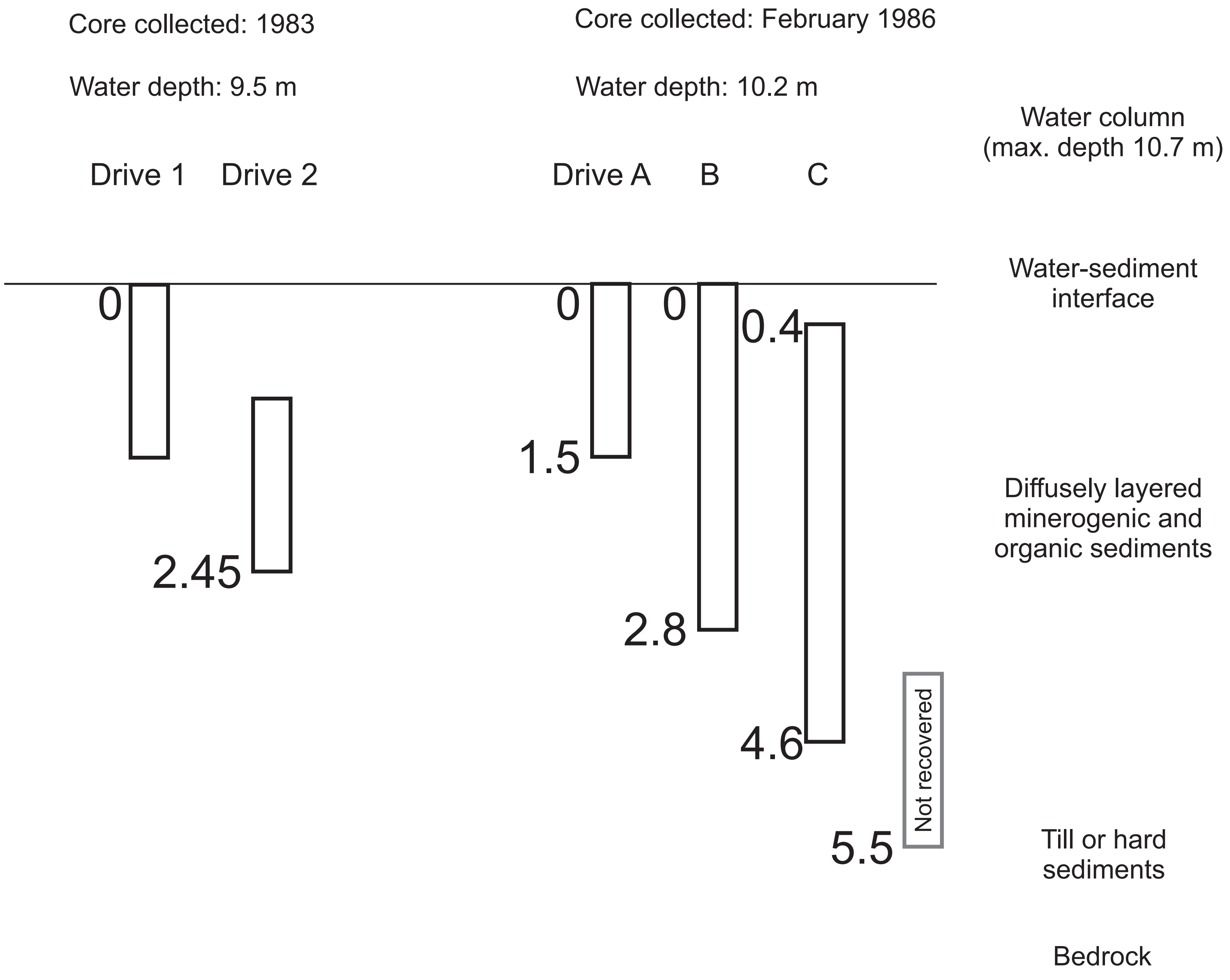

Supplement: S1 Fig — Sediment cores recovered during two fieldwork expeditions in 1983 and 1986. (PDF) [file pone.0184925.s001.pdf]
